# Editorial: Cyber security in the wake of fourth industrial revolution: opportunities and challenges

**DOI:** 10.3389/fdata.2024.1369159

**Published:** 2024-02-21

**Authors:** Elochukwu Ukwandu, Chaminda Hewage, Hanan Hindy

**Affiliations:** ^1^Cybersecurity and Information Network Centre, Cardiff School of Technologies, Cardiff Metropolitan University, Wales, United Kingdom; ^2^Department of Computer Science, Faculty of Computer and Information Sciences, Ain Shams University, Cairo, Egypt

**Keywords:** cyber-attack, Industry 4.0, data leaks, cyber-attack identification, cyber situational awareness, authentication

## Aims and objectives

This Research Topic focuses on eliciting knowledge through empirical evidence of the state of cyber security in the wake of the Fourth Industrial Revolution. The fourth industrial revolution (Industry 4.0) is inevitable as robust evidence exists to validate Industry 4.0, which harnesses physical, digital, and biological technologies. These disruptive and innovative technologies support enhanced efficiencies in processes and practices but equally face challenges in the level of risks with respect to cyber security because of the reliance on global system interconnections through the public internet and multi-tenanted cloud-based infrastructures. Industry 4.0 relies on high-speed internet connectivity, cloud computing, augmented reality, additive manufacturing, data science, and artificial intelligence for practical utilization and ease of deployment. This Research Topic provides an understanding of the changing dynamics and rapid proliferation in the automation of cyberattacks. The essence is to elucidate the opportunities that cybersecurity offers to mitigate the dynamics of cyberattacks perpetrated by highly sophisticated malicious actors. It also provides information on the disadvantages of using artificial intelligence and machine learning to automate cyberattacks.

Overall, the goal of this research is to elicit knowledge that investigates the opportunities brought about by the emerging Industry 4.0, such as smart cities, hospitals, transport, and digital health. In addition, this Research Topic provides existing knowledge and information on the trend of cyberattacks in these domains and the best practices for mitigating them. Furthermore, because Internet of Things (IoT) devices are one of the driving forces of automation in Industry 4.0, focusing on safe and secure deployment methods is important.

## Contributing articles

Four articles were published on this Research Topic.

### Article number one entitled: *What do you think about your company's leaks? A survey on end-users perception toward data leakage mechanisms*

This is an original technical article written by three authors, led by Bertrand, who conducted a survey to determine how employees perceive the prevailing data leak prevention mechanisms (Bertrand et al.). A survey was conducted with 150 employees using an online questionnaire and focused on deciphering the efficiency of existing data leak mechanisms. The results showed that these solutions lack usability and can be intrusive and blocking for employees. They also showed that employees prefer contextual, usable, acceptable, non-intrusive, and user-friendly anti-data leakage mechanisms within their organizations.

This article falls within the scope of the collections that focus on elucidating knowledge of the opportunities of Industry 4.0, as well as the attendant cyber security risks and countermeasures. The results will assist industry practitioners in focusing on using contextual, usable, and non-intrusive anti-data leakage mechanisms that will not disrupt employees' routine schedules by blocking their access to data required for effective day-to-day office activities.

### Article number two entitled: *Top-down machine learning-based architecture for cyberattacks identification and classification in IOT communication networks*

This is a single-author article led by Abu Al-Haija on a systematic review of machine learning-based architectures for detecting cyber-attacks in IoT communication networks. The results proposed an efficient and generic top-down architecture for intrusion detection and classification in IoT networks that use a non-traditional machine learning tool. The proposed architecture can be customized and used for intrusion detection/classification using any IoT cyber-attack dataset, such as the CICIDS and MQTT datasets.

In line with the topic of *Attacks targeting Internet of Things (IoT) devices, countermeasures, and secure methods of deployment*, the proposed architecture is promising. It can also provide new knowledge to researchers and practitioners on methods to mitigate network intrusion-based attacks.

### Article number three entitled: *A* 3D mixed reality visualization of network topology and activity results in better dyadic cyber team communication and cyber situational awareness

An original technical article by six authors, led by Ask, focused on studying tools to assist in cyber-defense decision-making (Ask et al.). This type of tool provides more efficient communication of cyber threat information between individuals in both educational and cyber threat situations. To this end, the study examined how the visual representation of network topology and traffic in 3D mixed reality vs. 2D affected teams performance in a sample of cybercadets (*N* = 22) cooperating in dyads.

The results of this study are consistent with the topic of *Predict the future of the cyber security scenarios and proactive countermeasure*. The results showed that participants who used 3D mixed-reality visualization had better cyber situational awareness than those in the 2D group and were better informed on ways of improving cyber situation awareness in an organization.

### Article number four entitled: *Authentication, access, and monitoring system for critical areas with the use of artificial intelligence integrated into perimeter security in a data center*

This was an original technical article written by two authors, led by Villegas-Ch and García-Ortiz. This study proposed using artificial intelligence in the perimeter security of data centers in addition to other physical security monitoring equipment, such as security cameras, movement detection systems, and authentication systems. The authors opined that integrating AI systems would provide for the automation and optimization of security processes, which translate into increased efficiency and reliability in operations that prevent intrusions through authentication, permitting verification, and monitoring critical areas. Furthermore, it is crucial to ensure that AI-based perimeter security systems are designed to protect user privacy. Overall, it is essential to regularly monitor the effectiveness and integrity of these systems to ensure that they function correctly and meet security standards.

This falls under the topic *Predict the future of the cyber security scenarios and proactive countermeasure* and shows how AI tools can be used to enhance and improve physical security monitoring in a data center. We are optimistic that physical security providers will find these results useful.

## Conclusion

The cybersecurity landscape has rapidly changed in recent years. Researchers and technologists have begun to focus on Industry 5.0 and quantum cryptography. New laws are being introduced to control the invasion of individual privacy by Artificial Intelligence (AI). For example, the EU AI Act will come into action soon and start categorizing AI applications, which could have privacy implications. Therefore, a joint approach between technical and policy interventions is required to curtail sophisticated cyberattacks on emerging applications and service offerings.

## Author contributions

EU: Conceptualization, Writing—original draft, Writing—review & editing. CH: Validation, Writing—review & editing. HH: Validation, Writing—review & editing.

